# Comparative performance evaluation of four commercial multiplex real-time PCR assays for the detection of the diarrhoea-causing protozoa *Cryptosporidium hominis/parvum*, *Giardia duodenalis* and *Entamoeba histolytica*

**DOI:** 10.1371/journal.pone.0215068

**Published:** 2019-04-08

**Authors:** Silvia Paulos, José María Saugar, Aida de Lucio, Isabel Fuentes, María Mateo, David Carmena

**Affiliations:** 1 Synlab Group, Microbiology Service, Quirón Madrid University Hospital, Pozuelo de Alarcón, Madrid, Spain; 2 Parasitology Reference and Research Laboratory, National Centre for Microbiology, Carlos III Health Institute, Majadahonda, Madrid, Spain; 3 European University of Madrid, Villaviciosa de Odón, Madrid, Spain; Aga Khan University - Kenya, KENYA

## Abstract

**Background:**

Multiplex molecular panels are relentlessly replacing conventional methods for the detection of enteric pathogens from stool samples in clinical and research laboratories. Here we evaluated four commercial multiplex real-time PCR assays for the detection of *Cryptosporidium hominis*/*parvum*, *Giardia duodenalis* and *Entamoeba histolytica*.

**Methods:**

The diagnostic performance of the Gastroenteritis/Parasite Panel I (Diagenode), the RIDAGENE Parasitic Stool Panel (R-Biopharm), the Allplex Gastrointestinal Parasite Panel 4 (Seegene) and the FTD Stool Parasites (Fast Track) real-time PCR methods was assessed against a reference panel of 126 well-characterized DNA samples including *Cryptosporidium hominis* (*n* = 29), *Cryptosporidium parvum* (*n* = 3), *Giardia duodenalis* (*n* = 47), *Entamoeba histolytica* (*n* = 3), other parasite species (*n* = 20), and apparently healthy subjects (*n* = 24).

**Principal findings:**

Obtained diagnostic sensitivities ranged from 53–88% for *Cryptosporidium hominis/parvum*, and from 68–100% for G. *duodenalis*. The R-Biopharm method achieved the best performance for the detection of *Cryptosporidium hominis/parvum* both in terms of diagnostic sensitivity (87.5%) and detection limit (a 100-fold increase compared to other tests). The Fast Track method was particularly suited for the detection of *G*. *duodenalis*, achieving a 100% sensitivity and a detection limit at least 10-fold superior. Detection of *E*. *histolytica* was similarly achieved by all compared methods except Diagenode.

**Conclusions:**

Diagnostic performance varied largely depending on the method used and the targeted pathogen species. Factors including test sensitivity/specificity, cost, patient population surveyed, laboratory workflow, and diagnostic algorithm should be carefully considered when choosing the most appropriate multiplex PCR platform.

## Introduction

Enteric *Giardia duodenalis*, *Cryptosporidium* spp., and *Entamoeba histolytica* are the most important diarrhoea-causing protozoa globally. Infections by these parasites cause significant morbidity and mortality primarily among children living in resource-poor settings in developing countries [1], but are also a significant public health concern in developed nations [2]. Indeed, these three protozoan species account for up to 70% of the gastrointestinal parasites diagnosed every year at hospital-based microbiology laboratories in Europe [3]. Additionally, both *G*. *duodenalis* and *Cryptosporidium* spp. are increasingly recognized as important waterborne and foodborne pathogens all over the world [4–6].

Light microscopy stands as the preferred routine diagnosis method for enteric protozoan parasites in most clinical settings. Although this technique is labour-intensive, lacks sensitivity, and requires skilled technicians, its simplicity and low cost outweighs the above mentioned limitations and makes microscopy suited for resource-limited laboratories particularly in endemic, high-prevalence areas. However, in high-income countries where parasite prevalence rates and burden are typically low and diagnostic sensitivity become an issue, a different diagnostic approach is clearly needed [7,8]. Other pressing issues include growing costs of labour, increased sample testing, a desire for improved throughput, and optimized laboratory workflows. All together, these facts explain why microscopy is being progressively replaced by highly sensitive DNA-based assays as first-line routine diagnostic methods for intestinal parasites in many clinical settings in western countries, mainly in Europe [8,9].

In recent years a wide diversity of in-house real-time PCR (qPCR) assays have been developed for detecting enteric viral, bacterial and parasitic, diarrhoea-causing, agents, with the trend fast moving from single pathogen detection to a multiplex approach allowing simultaneous identification of multiple pathogens [10,11]. An additional advantage of this technology is that it can be easily adapted to specific diagnostic requirements (e.g. pathogen combinations) depending on the population or patient group under study. Currently, several multiplex gastrointestinal pathogen panel tests are commercially available, including fully integrated robotic systems incorporating DNA extraction, amplification, detection, and analysis directly from stool samples [9,12]. A number of these methods (e.g. BD MAX Enteric Parasite Panel, Dickinson and Company, USA; Luminex xTAG Gastrointestinal Pathogen Panel, Luminex Corporation, Canada; NanoCHIP GIP, Savyon Diagnostics Ltd, Israel) have received clearance from public health agencies like the U.S. Food and Drug Administration (USA) and are being progressively incorporated in the diagnostic algorithms of modern clinical laboratories, particularly those attending large populations of paediatric, immunocompromised, or returning traveller populations [9]. Of notice, most of the studies conducted to evaluate the diagnostic performance of these and similar methods were based on prospectively and/or retrospectively collected stool samples with a previous diagnosis by microscopy examination [13–19]. In addition, a recent inter-laboratory external quality assessment scheme has evidenced the need of harmonization of molecular-based protocols and procedures for the detection of enteric protozoa within the European Union [20].

Here we aimed to evaluate the diagnostic sensitivity and specificity of four commercial multiplex qPCR assay for the specific detection of *Cryptosporidium hominis/parvum*, *Giardia duodenalis* and *Entamoeba histolytica*. The study was conducted against a reference panel of DNA samples extracted from stool specimens.

## Materials and methods

### Ethics statement

The study design and consent procedures involved in this survey have been approved by the Research Ethics Committee of the Carlos III Health Institute under reference number CEI PI 17_2017-v3. Written informed consent was not required for this study because the stool samples used were exclusively intended for routine clinical diagnostic procedures. All samples were anonymized using a unique laboratory identifier code to guarantee the anonymity and confidentiality of the patients.

### DNA reference panel

A total of 126 well-characterised DNA samples extracted and purified from stool specimens of clinically confirmed patients were obtained from a previously published study by our laboratory [21]. These included PCR-positive samples for *Cryptosporidium hominis* (*n* = 29), *C*. *parvum* (*n* = 3), *Giardia duodenalis* (*n* = 47), and *Entamoeba histolytica* (*n* = 3). No *Cryptosporidium* species other than *C*. *hominis* and *C*. *parvum* were assessed. Potential cross-reactivity was assessed against DNA samples positive for *E*. *dispar* (*n* = 10), *Leishmania infantum* (*n* = 2), *Trypanosoma cruzi* (*n* = 2), *Toxoplasma gondii* (*n* = 2), *Ascaris lumbricoides* (*n* = 3), *Strongyloides stercoralis* (*n* = 1), and apparently healthy subjects (*n* = 24) with a negative result for *Cryptosporidium hominis/parvum*, *G*. *duodenalis* and *E*. *histolytica*/*dispar* by PCR. Four aliquots of each individual DNA sample were prepared and stored at –20°C until tested with each assay to prevent degradation by freeze-thawing. The full dataset showing the characteristics of the DNA sample panel used and the diagnostic results obtained with each method is shown in S1 Table.

### Multiplex real-time PCR assays

The four commercial multiplex real-time PCR (qPCR) assays compared here were the Gastroenteritis/Parasite Panel I (Diagenode, Seraing, Belgium), the RIDAGENE Parasitic Stool Panel (R-Biopharm, Darmstadt, Germany), the Allplex Gastrointestinal Parasite Panel 4 (Seegene, Seoul, Korea), and the FTD Stool Parasites (Fast Track Diagnostics, Luxembourgh). The main features of these assays, including previously reported diagnostic sensitivity and specificity values, are shown in Table 1.

**Table 1:** Main features and reported diagnostic performance of the four commercial multiplex real-time PCR assays compared in the present study for the detection of *Cryptosporidium hominis/parvum*, *Giardia duodenalis*, and *Entamoeba histolytica* from clinical DNA samples.

Method	Manufacturer	Automated DNA extraction	Sensitivity (%)	Specificity (%)	References
Gastroenteritis/Parasite Panel I	Diagenode	No	92–100	100	[3,15]
RIDAGENE Parasitic Stool Panel	R-Biopharm	No	95–100^a^	99–100^a^	–
Allplex Gastrointestinal Parasite Panel 4	Seegene	Yes	NS	NS	–
FTD Stool Parasites	Fast Track	No	NS	NS	[13]

Notes: the RIDAGENE Parasitic Stool Panel (R-Biopharm) is designed to also detect *Dientamoeba fragilis*. The Allplex Gastrointestinal Parasite Panel 4 (Seegene) is designed to also detect *Blastocystis hominis*, *Dientamoeba fragilis*, and *Cyclospora cayetanensis*.

NS: not specified.

^a^ As reported by the manufacturer.

In an attempt to normalise initial experimental conditions among assays, DNA samples (5 μL for all methods excepting the FTD Stool Parasites method, for which 10 μL were used following the manufacturer´s recommendation) were tested undiluted in a 25 μL final volume. No sample duplicates were carried out. In the case of PCR inhibition, the sample was diluted 10-fold and retested. Experiments were performed on a Corbett Rotor-Gene 6000 qPCR cycler (Qiagen Corbett, Hilden, Germany) except otherwise indicated. All four methods included appropriate negative, positive, and qPCR inhibition controls. Multiplex qPCR protocols were conducted in strict accordance with the manufacturer´s instructions with few modifications as follows:

1. RIDAGENE Parasitic Stool Panel (R-Biopharm). Amplification reactions were conducted on an Mx3005P qPCR instrument (Agilent Technologies, Waldbronn, Germany) provided by the manufacturer as the method was not yet fully validated for the Rotor-Gene 6000 qPCR system at the time of the analyses.
2. Allplex Gastrointestinal Parasite Panel 4 (Seegene). This method includes an automated DNA extraction system that was not used in the present study for comparative reasons. Amplification reactions were carried out on the CFX96 qPCR instrument (Bio-Rad Laboratories, CA, USA) provided by the manufacturer.

### Simulated mixed infections

To mimic natural co-infections involving double (*Cryptosporidium* + *Giardia*) and triple (*Cryptosporidium* + *Giardia* + *E*. *histolytica* or *E*. *dispar*) pathogen combinations a total of 10 different simulated mixes were artificially generated. Each combination was prepared by mixing equal amounts of individual DNA samples from the reference panel (S1 Table). Simulated mixed infections were also used to indirectly assess diagnostic sensitivities and specificities of each method.

### Relative detection limit

Ten-fold serial dilutions of individual *Cryptosporidium* (undiluted to 10^−3^) and *Giardia* (10^−1^ to 10^−4^) positive DNA samples from the reference panel were tested in each compared method for detection limit determination based on cycle threshold (Ct) values obtained during qPCR. This assessment was not conducted for *E*. *histolytica* due to the low number of samples available and the high Ct values associated to them.

### Data analyses

The Shapiro-Wilk's test was used to assess the normality of distribution of the Ct values obtained in *Cryptosporidium*- and *Giardia*-positive samples during qPCR analyses with each method evaluated. Once normality was demonstrated, an analysis of variance (ANOVA) for simultaneous comparison of methods was conducted. A probability (*P*) value < 0.05 was considered evidence of statistical significance. Statistical analyses were performed using the software package SPSS version 21.0 (IBM Corp., USA).

## Results

**Fig 1:**
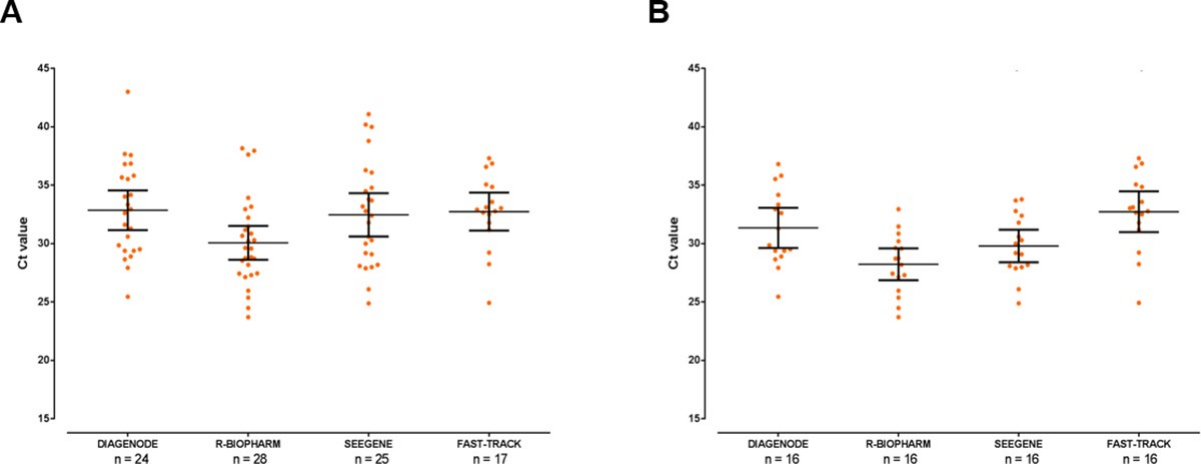
Dotplot showing the distribution of Ct values for *Cryptosporidium*-positive DNA samples obtained with each of the four commercial multiplex real-time PCR assays evaluated in this study. Mean values and standard deviation ranges for each group are represented by large and short horizontal bars, respectively. Unpaired (panel A) and paired (panel B) groups were represented for comparative purposes.

The diagnostic performance results of the four multiplex qPCR methods compared here are summarized in Table 2. The R-Biopharm method was the most sensitive (87.5%) assay for the detection of *Cryptosporidium hominis/parvum*, with the Fast Track assay performing poorly (53.1%). All four methods detected the three *C*. *parvum* DNA samples assessed. When tested undiluted, *Cryptosporidium*-positive DNA samples generated an elevated number of inhibitory reactions mostly resolved when re-tested in a 1:10 dilution. This was particularly true for the R-Biopharm and the Diagenode methods (of note, the former manufacturer specifically recommends diluting faecal suspensions 1:3 prior to DNA extraction). This issue was observed neither for *Giardia*- nor *Entamoeba*-positive DNA samples. One-way ANOVA test results showed significant (*P* < 0.05) variations in the distribution of obtained Ct values among the four multiplex qPCR methods assessed here, although no one-by-one direct comparison of methods was attempted due to unsurmountable differences in equipment (e.g. thermocycler used) features and assay (e.g. initial volume of DNA tested) procedures. In line with the findings mentioned above, the R-Biopharm method produced lower mean Ct values either in unpaired (mean: 30.1; 95% CI: 28.6–31.5) (Fig 1A) and paired (mean: 28.2; 95% CI: 26.9–29.6) (Fig 1B) *Cryptosporidium*-positive DNA samples than the other three methods tested.

**Table 2:** Diagnostic performance of the four commercial multiplex real-time PCR assays compared in the present study for the detection of *Cryptosporidium hominis/parvum*, *Giardia duodenalis*, and *Entamoeba histolytica* using a reference panel of well-characterized DNA samples (*n* = 126) from clinical specimens and healthy subjects.

		Diagenode		R-Biopharm		Seegene		Fast Track	
Protozoan pathogen	Samples (*n*)	+	%	+	%	+	%	+	%
*Cryptosporidium* spp.	32	24	75.0	28	87.5	25	78.1	17	53.1
*C*. *hominis*	29	21	72.4	25	86.2	22	75.9	14	48.3
*C*. *parvum*	3	3	100	3	100	3	100	3	100
*Giardia duodenalis*	47	32	68.1	37	78.7	43	91.5	47	100
*Entamoeba histolytica*	3	0	0.0	2	66.7	3	100	2	66.7
Other parasite species	20	0	0.0	0	0.0	0	0.0	0	0.0
*Entamoeba dispar*	10	0	0.0	0	0.0	0	0.0	0	0.0
*Leishmania infantum*	2	0	0.0	0	0.0	0	0.0	0	0.0
*Trypanosoma cruzi*	2	0	0.0	0	0.0	0	0.0	0	0.0
*Toxoplasma gondii*	2	0	0.0	0	0.0	0	0.0	0	0.0
*Ascaris lumbricoides*	3	0	0.0	0	0.0	0	0.0	0	0.0
*Strongyloides stercoralis*	1	0	0.0	0	0.0	0	0.0	0	0.0
Healthy subjects	24	0^a^	0.0	1^b^	4.2	1^c^	4.2	ND	ND

Notes: ´+´ refers to positive detection, ND stands for ´not determined´.

^a^ Only 13/24 samples tested.

^b^ Sample positive to *G*. *duodenalis* with a Ct value = 37.73.

^c^ Sample positive to *Cryptosporidium hominis/parvum*. with a Ct value = 40.20.

**Fig 2:**
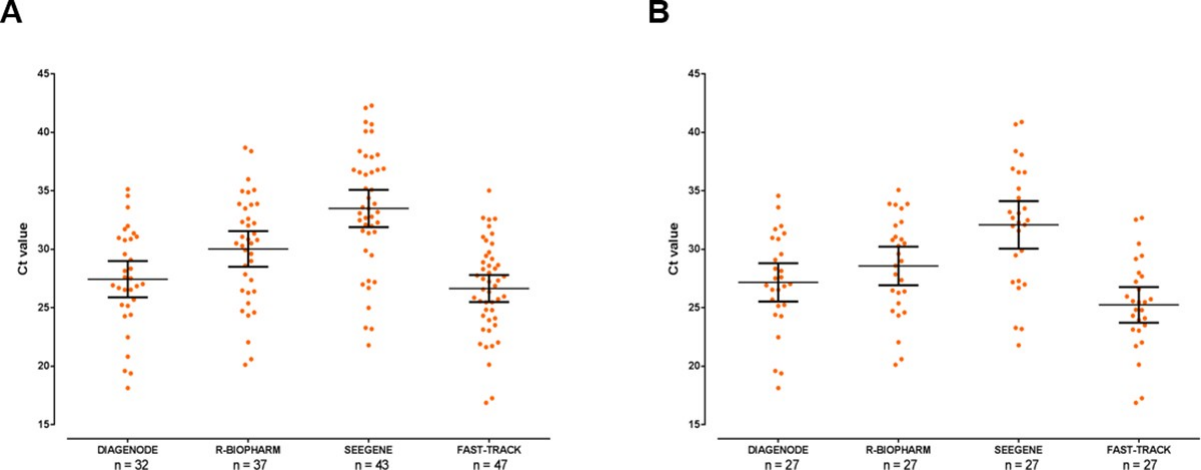
Dotplot showing the distribution of Ct values for *G*. *duodenalis*-positive DNA samples obtained with each of the four commercial multiplex real-time PCR assays evaluated in this study. Mean values and standard deviation ranges for each group are represented by large and short horizontal bars, respectively. Unpaired (panel A) and paired (panel B) groups were represented for comparative purposes.

Regarding *G*. *duodenalis*, the Fast Track method showed a 100% sensitivity, closely followed by the Seegene assay (91.5%). The latter method also detected all three *E*. *histolytica* positive samples, whereas the R-Biopharm and Fast Track produced a positive result for two out of these three samples. When Ct values for *G*. *duodenalis*-positive samples were plotted, the Fast Track method generated the lowest figures either in unpaired (mean: 26.6; 95% CI: 25.5–27.8) (Fig 2A) and paired (mean: 25.2; 95% CI: 23.7–26.8) (Fig 2B) sample groups. Very similar results were obtained with the Diagenode assay. No obvious association between *Giardia*-negative samples by the Diagenode, R-Biopharm, and Seegene methods and the magnitude of the qPCR Ct values at initial diagnosis (ranging from 20.7 to 32.8) was observed (S1 Table).

No non-specific amplification/cross-reactivity was seen in any of the four multiplex qPCR methods when DNA samples from other parasitic/commensal species (*E*. *dispar*, *L*. *infantum*, *T*. *cruzi*, *T*. *gondii*, *A*. *lumbricoides* and *S*. *stercoralis*) were tested. All 24 DNA samples from apparently healthy subjects tested negative, excepting one sample that was *G*. *duodenalis*-positive by R-Biopharm (Ct = 37.7) and a sample that was *Cryptosporidium*-positive by Seegene (Ct = 40.2). A number of previously unnoticed co-infections were observed within the panels of selected DNA samples positive for *Cryptosporidium hominis/parvum*, *G*. *duodenalis* and *E*. *histolytica*/*dispar*, most of them associated with high (≥ 35) Ct values (S1 Table). This finding is most likely due to the comparatively higher detection sensitivity of the multiplex qPCR methods with those of the conventional PCRs used in the primary diagnosis of the samples, although the occurrence of false-positive results could not be completely ruled out.

Testing of artificially-prepared mixed infections showed that, overall, the R-Biopharm method delivered the most consistent diagnostic results (Table 3). This method detected *Cryptosporidium hominis/parvum* in all simulated mixed infections except one, with lower Ct values than those obtained by the other three assays. Both the R-Biopharm and the Diagenode methods were also the only procedures able to detect *G*. *duodenalis* in all 10 mixed combinations assessed, even considering that the Fast Track system typically generated lower Ct values for this particular species. Additionally, detection of *E*. *histolytica* was only achieved by the R-Biopharm and the Fast Track methods in two out of the three samples tested, the latter assay showing the lowest Ct values.

**Table 3:** Diagnostic performance of the four commercial multiplex real-time PCR assays compared in the present study for the detection of *Cryptosporidium hominis*, *Giardia duodenalis*, and *Entamoeba histolytica* in artificially prepared DNA samples mimicking mixed infections. Cycle-threshold values are shown.

	*Cryptosporidium hominis*				*Giardia duodenalis*				*Entamoeba histolytica*			
Combination	Diagenode	R-Biopharm	Seegene	Fast Track	Diagenode	R-Biopharm	Seegene	Fast Track	Diagenode	R-Biopharm	Seegene	Fast Track
1: Cr + G + Eh	28.9	28.9	30.3	33.5	32.0	38.7	39.1	30.2	Neg.	41.8	Neg.	30.4
2: Cr + G + Eh	38.6	31.3	34.4	Neg.	20.9	25.7	25.0	19.4	Neg.	38.7	Neg.	33.4
3: Cr + G + Eh	Neg.	Neg.	Neg.	Neg.	24.9	29.5	28.2	23.7	Neg.	Neg.	Neg.	Neg.
4: Cr + G + Ed	37.6	29.1	33.4	Neg.	28.4	31.8	33.9	Neg.	–	–	–	–
5: Cr + G	26.7	25.9	27.0	34.2	23.8	27.8	27.7	29.4	–	–	–	–
6: Cr + G	28.0	27.5	28.6	Neg.	25.5	29.8	29.8	19.4	–	–	–	–
7: Cr + G	40.9	30.5	34.3	Neg.	31.5	32.6	Neg.	23.7	–	–	–	–
8: Cr + G	Neg.	37.7	Neg.	Neg.	26.8	29.6	30.1	26.8	–	–	–	–
9: Cr + G	Neg.	29.7	32.6	Neg.	32.5	39.1	38.3	21.9	–	–	–	–
10: Cr + G	39.9	30.6	34.0	Neg.	27.6	32.3	31.9	24.9	–	–	–	–

Notes: ´Neg.´ refers to a negative detection in the presence of parasitic DNA; ´–´ refers to a negative detection in the absence of parasitic DNA; Cr, *Cryptosporidium hominis*; G, *Giardia duodenalis*; Eh, *Entamoeba histolytica*; Ed, *Entamoeba dispar*.

**Fig 3:**
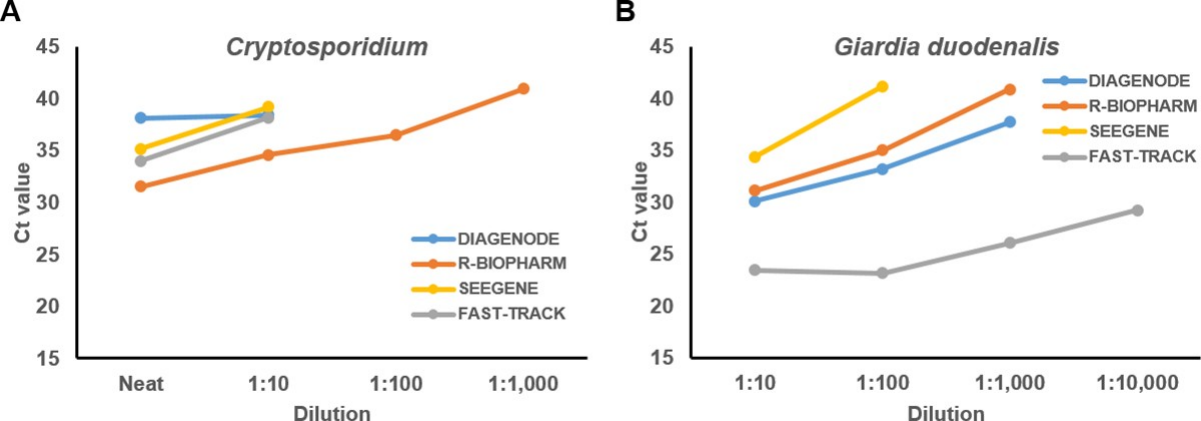
Relative detection limit analyses of the four commercial multiplex real-time PCR assays evaluated in this comparative study using serial dilutions of *Cryptosporidium*-positive (panel A) and *Giardia*-positive (panel B) individual DNA samples.

Confirming previous diagnostic findings, relative detection limit analyses based on serial dilutions of one sample positive for *Cryptosporidium* and one sample positive for *G*. *duodenalis* demonstrated that the R-Biopharm method was two order of magnitude more sensitive for the detection of *Cryptosporidium* than the other three assays evaluated (Fig 3A). For the detection of *G*. *duodenalis*, the best performance was achieved by the Fast Track method, at least one order of magnitude more sensitive than that of the other assays (Fig 3B).

## Discussion

In this comparative study we evaluated four commercial multiplex qPCR methods for the identification of the three most clinically relevant protozoan enteric parasites, namely *Cryptosporidium* spp., *Giardia duodenalis*, and *Entamoeba histolytica*. The diagnostic performance of both the Diagenode and the Fast Track assays have been individually assessed and compared to that obtained by microscopy in previous studies [3,13,15]. However, this is the first comprehensive survey reporting the diagnostic performance of the R-Biopharm and Seegene methods. Another significant methodological contribution of this work is that, in an attempt to normalize starting experimental conditions and strengthen the robustness of the obtained data, analyses were conducted against a reference panel of well-characterize DNA samples. Our results evidenced marked detection differences among tests depending on the targeted parasite species considered, with diagnostic sensitivities typically ranging from 53–88% for *Cryptosporidium* spp., and from 68–100% for *G*. *duodenalis*. The diagnostic sensitivity of these methods for the detection of *E*. *histolytica* was not fully assessed due to the insufficient number of positive samples to this pathogen available. Despite the highly variable sensitivity values observed, all four methods did not cross-react with any of the DNA samples from other protozoan or helminthic parasite species tested, suggesting a specificity near 100%. However, the exact extent of this statement should be confirmed in future studies including a larger panel of DNA samples from other potentially cross-reacting enteric pathogen and commensal species including *Cyclospora cayetanensis*, *Entamoeba coli*, *Endolimax nana*, *Blastocystis* sp., and *Dientamoeba fragilis*.

Regarding *Cryptosporidium* detection, the R-Biopharm method achieved the best performance in terms of diagnostic sensitivity (87.5%) and detection limit (two order of magnitude superior to that of the other tests). In contrast, the Diagenode (diagnostic sensitivity: 75.0%) and the Fast Track (diagnostic sensitivity: 53.1%) assays performed comparatively worse. This finding is hardly surprising when considering that both methods at best equalled the diagnostic performance of conventional microscopy when attempting to detect *Cryptosporidium* in clinical samples [3,13]. However, the Fast Track method was much better suited for the detection of *G*. *duodenalis* than the other three assays evaluated here, achieving a 100% sensitivity and a detection limit at least one order of magnitude superior to that of the other three methods. This is well in agreement with previous findings in patients with diarrhoea from community or hospital sources, where the Fast Track assay detected 83% more *G*. *duodenalis*-infected cases than microscopy examination [13]. In contrast, the Diagenode method achieved the lowest diagnostic sensitivity (68.1%) for the identification of *G*. *duodenalis* of all four assays compared here. This finding is also in line with previous data demonstrating that this technique performs only moderately better than conventional microscopy during routine examination of clinical stool samples [3,15]. Regarding *E*. *histolytica*, all methods tested, excepting Diagenode, performed similarly well for the detection of *E*. *histolytica*, with the Fast Track assay providing the highest diagnostic sensitivity values. However, these figures should be interpreted with caution due to the comparatively low number of *E*. *histolytica* DNA samples available for the analyses. Considering all the above, our data demonstrated large differences in the diagnostic performance of the compared methods. In addition to the test sensitivity/specificity evaluated here, other factors including cost, patient population surveyed, laboratory workflow, and diagnostic algorithm should be carefully considered when choosing the most appropriate multiplex PCR platform for the simultaneous detection of *Cryptosporidium* spp., *G*. *duodenalis*, and *E*. *histolytica* in human stool samples.

Our study has some limitations. First, the reference panel contained a restricted number of DNA samples, particularly those positive to *E*. *histolytica* and to other intestinal pathogen and commensal species. This fact may have hampered the accuracy of some of the obtained results. In order to minimize this issue, statistical analyses were based on the Shapiro-Wilk's test as non-parametric test, a method particularly suited for small to moderate samples sizes. Other potential confounding factors are the design of the primer sets used to amplify the selected targets and the use of different qPCR instruments depending on the method evaluated.

Accurate and fast detection of clinically relevant enteric protozoa is highly desirable for prompt, adequate, and effective treatment [10]. In this context, multiplex qPCR platforms have been reliably shown to increase the positivity rates of protozoan pathogens compared to conventional methods [13,22–24]. Even more importantly, multiplex molecular panels including viral, bacterial, and parasitic pathogens enable the syndromic testing of patients with gastrointestinal symptoms in a cost-effective manner [25]. Taken together, these facts explain why these methodologies are being increasingly used in diagnostic laboratories and reference centres. However, the choice of the most suitable assay should be based on a careful evaluation of variable including optimal laboratory workflow and diagnostic algorithm, test performance, and patient population to be tested.

## Supporting information

S1 Table Full dataset showing the characteristics of the DNA sample panel used, the initial experimental conditions, and the diagnostic results obtained with each method compared in the present study. (XLSX)
